# Electronic Noses and Their Applications for Sensory and Analytical Measurements in the Waste Management Plants—A Review

**DOI:** 10.3390/s22041510

**Published:** 2022-02-15

**Authors:** Justyna Jońca, Marcin Pawnuk, Adalbert Arsen, Izabela Sówka

**Affiliations:** 1 Department of Environment Protection Engineering, Faculty of Environmental Engineering, Wroclaw University of Science and Technology, Wybrzeże Wyspiańskiego 27, 50-370 Wrocław, Poland; justyna.jonca@pwr.edu.pl (J.J.); marcin.pawnuk@pwr.edu.pl (M.P.); 2 calval.pl sp. z o.o., Emili Plater 7F/8, 65-395 Zielona Góra, Poland; info@calval.pl

**Keywords:** electronic nose, machine learning, gas sensors, monitoring networks, olfactometry, GC-MS, odor impact assessment, waste management plants

## Abstract

Waste management plants are one of the most important sources of odorants that may cause odor nuisance. The monitoring of processes involved in the waste treatment and disposal as well as the assessment of odor impact in the vicinity of this type of facilities require two different but complementary approaches: analytical and sensory. The purpose of this work is to present these two approaches. Among sensory techniques dynamic and field olfactometry are considered, whereas analytical methodologies are represented by gas chromatography–mass spectrometry (GC-MS), single gas sensors and electronic noses (EN). The latter are the core of this paper and are discussed in details. Since the design of multi-sensor arrays and the development of machine learning algorithms are the most challenging parts of the EN construction a special attention is given to the recent advancements in the sensitive layers development and current challenges in data processing. The review takes also into account relatively new EN systems based on mass spectrometry and flash gas chromatography technologies. Numerous examples of applications of the EN devices to the sensory and analytical measurements in the waste management plants are given in order to summarize efforts of scientists on development of these instruments for constant monitoring of chosen waste treatment processes (composting, anaerobic digestion, biofiltration) and assessment of odor nuisance associated with these facilities.

## 1. Introduction

Waste management plants are well known for their significant impact on environment and human lives [1]. Processes related to management, treatment and disposal of wastes are potential sources of harmful compounds emissions into the water, soil and air [1,2,3]. Concerning emissions into the air following substances may be considered as a threat: CO, CO_2_, CH_4_, NH_3_, NO_x_, H_2_S, HCl, HF, dioxins, furans, PAHs and VOCs [3,4]. Many of these compounds are odorants and literature shows that their emission is one of the most significant problems associated with waste management plants. Indeed, prolonged exposure of inhabitants living in the neighborhood of this type of facilities can deteriorate their living quality by causing odor nuisance [5,6,7,8]. Moreover, increase in the amount of generated wastes, resulting from the development of societies, will cause growth in number of waste management plants and thus, intensification of waste treatment and disposal processes [2,5,6,9]. As a result, more people will be exposed in the future to excessive emission of odorants caused by waste management.

**Figure 1:**
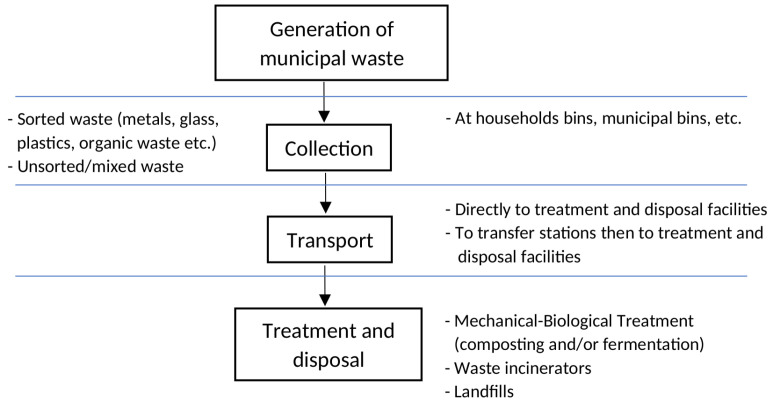
Simplified scheme of waste management system, based on [10,11,12,13].

As shown on Figure 1, the waste management system is based on several steps and each one of them can be a potential source of odorants, i.e., collection, transport, treatment and disposal of wastes (mechanical-biological treatment facilities, composting plants, landfills, waste incinerators) [10,11,12,13]. The first step in the waste management chain is the collection of wastes by residents at households and residential areas and their transport by specialized vehicles to the adequate facilities for treatment and disposal. Collected wastes can be transported directly to their final destination or temporary stored at a waste transfer station. Among the disposal and treatments methods the most advanced technologically is the mechanical-biological treatment (MBT) of wastes. The main purpose of the MBT is to recover useful materials (e.g., biogas, compost) and reduce the amount of wastes directed for landfilling. The MBT facilities are probably the most important odor sources along the waste treatment chain, mainly due to the employment of biological methods of waste decomposition, i.e., composting (aerobic treatment) or/and fermentation (anaerobic treatment). The latter process is crucial for emission of odorous substances [2,7,9]. Moreover, chemical composition of those volatile mixtures can vary depending on the type of waste being processed, the stage of waste decomposition, the selected treatment method and its operating conditions, i.e., temperature, humidity or aeration [4,10,14]. The way that every step in waste management system is carried out can vary on national, regional and local scales [13].

Literature provides information on the chemical composition of odorous mixtures emitted from different stages of waste management (Table 1). Those odorants can be divided into several chemical groups, i.e., sulfur compounds, nitrogen compounds, hydrocarbons (unsaturated and saturated), aromatics, terpenes, halogenated compounds, volatile fatty acids, carbonyls, and alcohols. The variety of these substances as well as the complexity of waste management processes constitute a serious challenge in the monitoring of emitted odorants and odors, which requires two different but complementary approaches: analytical (e.g., GC-MS, gas sensors) and sensory (e.g., dynamic olfactometry), respectively [15].

In dynamic olfactometry the determination of odor concentration, expressed in ou/m^3^, is achieved by presenting a sample to a panel of trained persons according to specific procedures (e.g., PN-EN 13725) [16]. However, information about its chemical composition can not be delivered from these measurements. On the contrary, GC-MS method gives concentrations of individual odorants, expressed in ppb or ppm, but this tells us very little about the perceived odor or interactions between odorants in the investigated mixture [17,18,19]. An interesting approach is to combine GC-MS with a sniffing port (GC-O-MS) so each chemical component of the analyzed odor is led to a human nose for evaluation. However, still, information about the total odor concentration or odorants complicated interactions can not be achieved with this method [20,21].

Single gas sensors are relatively cheap and simple to use [32]. For the waste management plants specific analyses of H_2_S and NH_3_ are of high interest. However, odorous samples are usually composed of hundreds of different molecules and since, it is difficult to fabricate selective sensors for each one of them, this method may not take into account all prevailing substances. Multi-sensor arrays can cover wide spectrum of substances due to the unspecific sensors. After training with the GC-MS technique they have the potential to continuously provide information on the chemical composition of odorous samples. Moreover, training with olfactometry measurements is also possible. The measured odor concentrations are in this case expressed in ou/m^3^ [33]. Both, single gas sensors and multi-sensor arrays can be applied on site for the constant monitoring of air quality [34]. Construction of an EN is a challenging task and can be affected by many factors, mainly associated with the selection of sensors, development of models and quality of data used for their training.

Table 2 summarizes the sensory and analytical measurement methods. It is hard to compare these techniques since each one of them provide different type of informations. Therefore, choice of the appropriate method has to be made on a case by case basis depending on the specific needs. An advanced way to analyze odorous substances is to combine olfactometry, GC-MS and EN as complementary approaches to a given task and thus, obtain maximum informations on the analyzed samples [35].

The next sections provide description of different methods discussed briefly above. Olfactometry, GC-MS, GC-O-MS and single gas sensors are presented in Section 2 and Section 3. Examples of applications of these techniques to the waste treatment plants are given.

Electronic noses are the core of this review and are described in details in Section 4. A special attention is given to the gas sensors used for EN systems construction. Indeed, different types of detectors are discussed and current trends in sensitive layers development are described. Additionally, relatively new EN based on flash GC technology are considered as recent studies showed that they are suitable for monitoring of odorous samples from landfills [36]. Data pre-processing and classification methods are addressed as well. Algorithmic solutions to common problems associated with EN (e.g., signal drift, ambient temperature changes) are given and current challenges in data processing are discussed. At the end of this section examples of commercially available EN are listed with special attention to devices dedicated to the odorous samples investigations. Although several reviews on EN has been published recently [37,38,39], they focus usually on devices made of gas sensors. We took into account EN based on other technologies and compared them with classical approach.

Examples of applications of EN for analytical and sensory measurements in the waste management plants are given in Section 5 and Section 6. A few review papers summarize efforts of scientists on development of EN for odor impact assessment [39,40,41]. Although, examples of applications of these devices to the waste treatment plants appeared in these papers, they represented just a small part of the discussion. The present survey focus solely on EN used for the odor impact assessment in the vicinity of these facilities and thus, fully exhaust the topic.

Some authors tried also to review the problem of different processes monitoring using, among many others, EN, e.g., anaerobic digestion [42] or composting [43]. Once again EN represented just a small part of a bigger picture and thus, only few examples of application of these devices were listed in mentioned papers. Our review focuses on EN development for the monitoring of the mechanical-biological treatment of waste and presents numerous examples of applications of these devices for the investigation of composting, anaerobic digestion or bio-filtration processes.

In summary, this review describes efforts of scientists on development of EN and their applications for odor impact assessment in the vicinity of waste management plants and monitoring of odorants emitted during mechanical–biological treatment of waste. Therefore, the paper presents two approaches to the problem of the odorous mixtures investigation with electronic noses: a sensory and analytical one.

## 2. Sensory Measurements

### 2.1. Dynamic Olfactometry

Sensory measurements employ a panel of human noses in conjunction with the olfactometer–an instrument which dilutes analyzed sample with odor-free air, in order to determine odor concentration. Two standardized methods of sample presentation to the panel are applied: forced choice and yes/no method. In the first one two sniffing ports are used but the odor sample is presented only at one of them. The examiner has to choose the port from which she/he perceives the odor. In the second method examiner sniffs from a single port and communicates if an odor is detected or not [16,44,45].

Odor mixtures at different dilutions are presented to examiners for sniffing usually in an ascending order. The process continues until each person positively detects an odor in the diluted mixture which means that the panel has reached the detection threshold for that odor [16]. The threshold is calculated as the geometric mean between the dilution of the last negative answer and the dilution of the first positive answer. The odor concentration (*C*) is expressed as the dilution required for achieving the panel detection threshold and can be described with the equation:

(1) $$C=\frac{V_{od}+V_{f}}{V_{od}}$$

where *V_od_* is the volume of odorous sample and *V_f_* is the volume of air required to reach the threshold. By analogy, for a dynamic olfactometer the concentration is given by:

(2) $$C=\frac{Q_{od}+Q_{f}}{Q_{od}}$$

where *Q_od_* is the flow of odorous sample and *Q_f_* is the flow of odor-free air required to reach the threshold. The concentration is usually expressed in ou/m^3^ [16].

Since the sensitivity to odors is variable among people, the examiners could measure different odor concentrations for the same sample. This effect is minimized by a careful selection of assessors with average olfactive sensitivity according to a standardized procedure [16]. The selection of examiners is performed using a reference odorant, usually n-butanol. Only persons with average n-butanol odor threshold in the range of 20–80 ppb and the antilog standard deviation for individual responses less than 2.3 are selected. Examiners must be continuously tested, trained and obey a simple behavior code (e.g., no smoking before measurements, exclusion from measurements during illness caused by cold, etc.) [16].

The panel referability to the standard odorant and the coherence of panel responses determine the quality of olfactometric measurements. In order to guarantee the referability, the laboratory performances are evaluated by accuracy and precision measures. The coherence of panel results is assured by a validation procedure that indicates panel member who gives invalid responses. If the threshold value given by an examiner does not meet certain criterion, then all measures provided by this person must be eliminated [16].

Except for the odor concentration other parameters can be measured with olfactometry as well, namely odor intensity and hedonic tone. Perceived odor intensity is the relative strength of the odor above its detection threshold. The intensity is usually represented by some kind of a category scale (i.e., weak, strong), by subjective magnitude estimates (i.e., odor X is twice as strong as odor Y) or by reference to a specific odorant, whose concentration is adjusted until both, the reference and analyzed sample have the same perceived intensity [46]. Usually, the panelists must assess the odor intensity of the sample according to a specified category scale. The most commonly applied scale counts seven categories from “no odor” to “extremely strong odor” [47].

Odor concentration and intensity are related. The perceived intensity increases with increasing odor concentration. Two models have been proposed to explain relation between those two parameters. The Weber–Fechner one produces a linear plot of intensity against log concentration:

(3) I=a·logC+b

Steven’s law turns-out a linear plot of log intensity against log concentration:

(4) $$I=k\cdot C^{n}$$

where *I* is the intensity, *C* the odorant concentration and *a*, *b*, *k*, *n* are the constants. The choice of the model depends on the representation of odor intensity. When category scale is used, the Weber–Fechner law shall be applied. When magnitude or reference scales are used, Steven’s law gives better results [46].

Hedonic tone defines the pleasantness and unpleasantness of an odor and is evaluated according to a category scale ranging from −4 (extremely unpleasant) through zero (neither pleasant nor unpleasant) to +4 (extremely pleasant) [47]. The hedonic tone and intensity can be used as parameters for odor impact and odor annoyance assessment by residents living in the neighborhood of industrial activities [48].

Dynamic olfactometry is used to characterize odor emission sources and requires collection and transportation of the samples to the laboratory. For this reasons this technique is not sufficient to asses the odor impact on citizens living in the vicinity of an odor source. Therefore, the application of dynamic olfactometry requires combination with dispersion modelling [49,50]. These mathematical models merge olfactometry results with meteorological and geographical data and calculate how the emitted odor is transported through the air to the people living in the neighborhood of a particular odor source [51,52].

### 2.2. Field Olfactometry

The field olfactometer is a portable device that allows to evaluate odors concentrations on site which is the main advantage of this technique as compare to the dynamic olfactometry [53]. A field olfactometer creates a series of dilutions by mixing the odorous ambient air with odor-free (carbon-filtered) air. Number of dilutions needed to make the odorous ambient air non detectable is defined as the Dilution-to-Threshold ratio [54]. Therefore, field olfactometry does not provide directly a measure of the odor concentration in ou/m^3^. Disadvantages of this technique include: odor fatigue (it is difficult not to expose the examiner to the odorous environment before the olfactometer is actually used), lack of dilution options and inability to test examiners with a reference odorant. Besides, it is difficult to remain objective when seeing sources of odor emissions.

### 2.3. Examples of Application of Sensory Measurements to the Waste Management Plants

Olfactometry is widely used for odor impact assessment in the vicinity of waste management plants. For example, a facility characterized by nine point and one area (landfill) odor sources was investigated recently [52]. Odor concentrations were measured by dynamic olfactometry and dispersion modelling was performed for two scenarios. In the first one both, the area and point emission sources are taken into consideration. Second scenario was conducted only for the point sources. It was concluded that the landfill is mainly responsible for odor nuisance caused by the plant and that closing this particular odor source will significantly improve air quality nearby. Indeed, dynamic olfactometry in combination with dispersion models is a useful tool for the optimization of odor mitigation strategies [55], but a great care must be taken when developing these models as recent studies suggest that modeling choices may lead to a variance in the resulting odor concentrations at receptors differing by up to a factor 3 [56].

Field olfactometry measurements can also be used as input variables in odor distribution modeling. The application of this method was demonstrated recently on a case study of a municipal landfill [53]. Moreover, local planning strategies based on odor dispersion modelling were proposed in order to improve air quality in the vicinity of the facility.

Gutiérrez et al. [57] showed that dynamic olfactometry may be used not only to feed dispersion models, but also to monitor composting process. Authors measured odor concentrations emitted during the composting of organic fraction of municipal solid wastes. Good correlation between odor concentration and traditional (i.e., physical-chemical and respirometric) variables was achieved. Moreover, olfactometric detection limit and the compost respirometric stability were reached simultaneously, suggesting that olfactometry is a sufficient and simple method to assess compost stability.

Dynamic olfactometry may also be used as a tool for evaluation of the efficiency of purifying odorous gases by bio-filtration [58,59]. Measurement of concentrations of individual pollutants by means of GC-MC does not always allow to assess the degree of olfactory pollution. Therefore, dynamic olfactometry is a very useful tool for this purpose since it allows to determine the degree of total deodorization efficiency. Efficiency of two filters, i.e., mineral and organic one was compared recently. Odor reduction of respectively 60.8% and 97.2% was achieved. An important aspect of this research was the evaluation of hedonic quality of analyzed samples since organic filters themselves are characterized by an intense natural odor [58].

Wiśniewska et al. [60] investigated odor nuisance in the close vicinity of a biogas plant by means of field olfactometry. Obtained results were compared with H_2_S and (CH_3_)_2_S analytical concentrations measured by portable GC-PID detector. Odor concentration values were in line with odorants content in the investigated samples and strongly related to the concentration of H_2_S. Authors noted also a strong relationship between odor nuisance, technological process used in the plant and the type of treated waste. These results suggest that field olfactometry is an appropriate method of process control carried out in biogas plants. Presented above applications are summarized in Table 3.

## 3. Analytical Measurements

### 3.1. GC-MS

Identification of individual odorants is difficult without using a separation technique. Therefore, gas chromatography is frequently applied for this task, and is often coupled with mass spectrometry (GC-MS). The latter allows identification and quantification of the species present in the analyzed sample. Since level of some odorants can be as low as ppb or even ppt, the pre-concentration techniques are commonly used before the actual analysis (e.g., adsorption of the odorants on active carbons followed by thermal desorption) [61]. The GC-MS technique gives informations that are fundamental to evaluate the impact of emitted compounds on the environment and human health. In the same time, GC-MS measurements are expensive, time-consuming and not suitable for on site applications.

It is also very difficult to relate the chemical composition of an odorous mixture to its odor concentration. One approach is to calculate the odor activity value (*OAV*) defined as the sum of the ratio between the chemical concentration of each compound in the mixture and its odor threshold concentration [41]:

(5) $$OAV=\sum_{i=1}^{n}\frac{C_{i}}{OT_{i}}$$

where *OAV* is the odor activity value (ou/m^3^), *C_i_* is the concentration of the compound *i* (mg/m^3^) and *OT_i_* is the odor threshold of the compound *i* (mg/ou).

Indeed, this method allows to estimate the odor concentration in the case of samples containing few odorants but real samples are usually very complex and odor concentrations calculated through *OAV* can be highly imprecise [62,63]. One source of misleading results is the difficulty of obtaining reliable odor threshold values. Sometimes literature screening gives *OT* values for a given odorant that differ by several orders of magnitude [64]. Moreover, different types of interactions between compounds may occur (synergic or masking effects), resulting in odors that can be hardly related to the chemical composition [65].

### 3.2. GC-O-MS

GC-O-MS combines two techniques: olfactometry and gas chromatography coupled with mass spectrometry. After separation the eluted flux is divided between the MS detector and the olfactory port for the chemical and sensory evaluation, respectively [66,67]. This technique provides detailed information on individual odorants presented in the analyzed mixture and allows to estimate their relative influence on the total odor of the sample. In the same time, the olfactory properties of the sample as a whole can not be obtained. Therefore, it is not possible to determine the odor concentration or provide information about the odor impact using this method. Similarly to the GC-MS, the GC-O-MS measurements are expensive, time-consuming and not suitable for on site applications [41].

### 3.3. Single Gas Sensors

Selective detection of certain odorants can be achieved through gas sensors. Devices based on electrochemical principle of detection are well suited for this task [68]. A typical electrochemical sensor set–up consists of three electrodes: the working, the reference and the counter electrode. The operating principle of an electrochemical sensor is based on the measuring current flow changes generated by reduction or oxidation of target molecules at the working electrode surface. The changes in the current flow are proportional to the measured gas concentration [69]. This type of sensors are usually highly selective and quite sensitive. However, there is a limited number of molecules that can be measured using this technique (e.g., SO_2_, NO_2_, NH_3_, H_2_S, HCHO, glutaraldehyde) [32].

A very important type of gas sensors are nondispersive infrared detectors (NDIR). The sensor set–up consists of a source of infrared radiation in alignment with a detector. When an analyzed gas enters the measurement chamber, it absorbs radiation of a particular wavelength causing decrease in light intensity reaching the detector. This reduction of infrared radiation is proportional to concentration of target molecules. An important element of the sensor is an optical filter, which passes absorbed light of defined wavelength, thus providing selectivity of particular sensor [70]. Similarly to the electrochemical sensors, the NDIR detectors are highly selective and can be used for CH_4_, CO and CO_2_ measurements. Although, non of these molecules can be considered as an odorant, the monitoring of methane content is of high interest when investigating, for example, the stability of the composting pile [71].

Specific gas sensors mentioned above are often applied together with electronic noses or even integrated to the construction of the device in order to increase its selectivity and provide complementary informations on the analyzed samples (e.g., Olfosense from Airsense).

The photoionization detector (PID) is another type of sensors that is often applied during the investigation of odorous samples. PID detects molecules that are ionized under the influence of radiation emitted by the UV lamp (their ionization energy is lower than the energy of the emitted photons). The presence of the ionization products is recorded by an electrometer [32]. Contrary to the electrochemical and NDIR detectors, PID ones are not selective. However, these sensors provide informations about the total amount of VOCs present in the analyzed samples and thus, can be a valuable addition to the multi-sensor arrays (e.g., Olfosense from Airsense).

### 3.4. Examples of Applications of Analytical Measurement to the Waste Management Processes Monitoring

GC-MS is a very powerful technique for the monitoring of odorants emitted during different stages of the waste management chain. Liu et al. [7] investigated recently VOCs released during initial decomposition of municipal solid waste, that occurs during waste collection, transportation and early pre-treatment. Ethanol was the dominant compound in these samples and identified as one of the main odorants (together with methyl sulfide, dimethyl disulfide and ethyl acetate). Moreover, authors calculated the odor activity value (OAV) following Equation (5) mentioned above. The easily biodegradable waste (EBW) proportion in waste is the dominate source of VOCs generation. Indeed, when the proportion of EBW was reduced from 60% to 15%, the OAV decreased from 244.51 to 61.46. However, validation of obtained results with dynamic olfactometry was not performed.

Composting can also be a source of odorous molecules and the waste origin plays a crucial role on the chemical composition of those substances. Sulfides, followed by acid/esters, ketones, alcohols, and terpenes are produced during composting of food wastes whereas terpenes, followed by aromatic hydrocarbons, ketones and alkanes are emitted from yard wastes. Addition of paper to the yard or food wastes results in formation of aromatic hydrocarbons and alkanes at the beginning of the composting process [72]. Similar VOCs were detected by Agapios et al. [73] during household composting of food wastes. Moreover, Principal Component Analysis (PCA) was also applied to explore the entire data set, including VOCs and physicochemical parameters for monitoring of the composting process in terms of compost maturity and biological activity. Indeed, emission of terpenes during the composting was related to the immature phase of the process, whereas high content of sulfides was associated with intense biological activity.

Single gas sensors are also widely used for the monitoring of odorants emitted during different waste treatment processes. Electrochemical ammonia sensor was used for the evaluation of the efficiency of NH_3_ removal from composting gases by bio-filtration [74]. Municipal solid wastes, digested wastewater sludge and animal by-products were composted in a pilot-scale reactor and the exhaust gas was treated in a biofilter. The NH_3_ removal efficiency of 95.9% was obtained in the described experiment for the first two waste sources but declined significantly in the case of animal by-products.

Wiśniewska et al. [75] used a portable multi-gas analyzer, MultiRae Pro, equipped with one PID detector and three electrochemical sensors to measure H_2_S, NH_3_ and methanethiol for the monitoring of odorants emitted from biogas plants. The highest concentrations of odorants were associated with oxygen stabilization of digestate (VOCs, NH_3_) and with technological wastewater generated at biogas plants (mainly NH_3_). Authors suggested that, the detector can be used to control technological processes by measuring the odorant concentrations and calculating the odour activity value (OAV). Based on the results, it should be possible to plan the activities aimed at minimizing the odour nuisance related to the presence of specific compounds in the process gases. However, validation of proposed method with dynamic olfactometry was not performed.

Mabrouki et al. [76], proposed a system to monitor the biogas generated from a landfill placed near the Morocco City and update the database remotely. The Internet of Things system consisted of Arduino Uno R3 Card, Wi-Fi module and Bluetooth module and set of gas sensors (CH_4_, CO_2_, CO, O_2_, NO_2_ and H_2_S) was used for this purpose. Biogas emitted form the landfill was rich in methane and carbon dioxide, but the exact content of the analyzed samples depended strongly on the season (water content) and the waste deposit age. Presented above applications are summarized in Table 4.

## 4. Electronic Noses

### 4.1. General Principle

**Figure 2:**
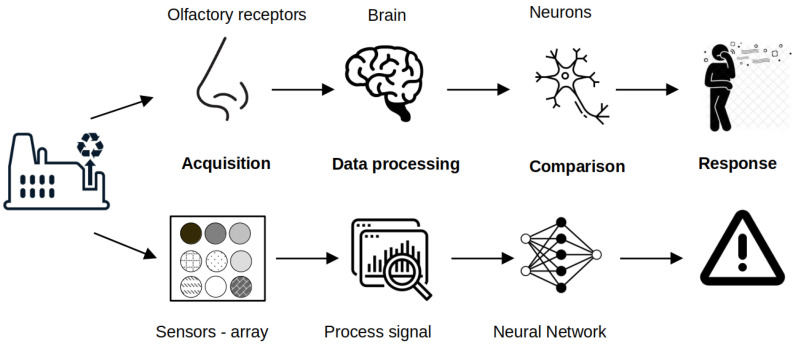
Structures of biological olfactory system and electronic nose.

The term “electronic nose” comes from some similarities between the analysis of volatile compounds in air using a set of gas sensors and the human olfactory system. Upon being sniffed through the nose, inhaled air reach the olfactory epithelium located in the upper nasal cavity [77]. Interactions of odorous molecules with the olfactory receptors produce electrical stimuli which are transmitted to the brain. There, a pattern recognition process assisted by the memory takes place in order to identify, classify and perform an hedonic analysis of the particular smell [78]. A single olfactory receptor responds usually to several odorants and each odorous molecule can interact with multiple olfactory receptors [79]. Similarly, working principle of the EN is based on the cross-reactivity and semi-selectivity of the gas sensors used to design the sensors array. The interactions of volatile compounds with these sensors give rise to analytical signals which are then processed by the computer via a pattern recognition program. Just like humans, EN can learn new patterns and associate them with new odors via training and data storage. The working principle of human and EN is presented on Figure 2.

Operationally, an EN is composed of three parts: a sampling system, an array of chemical gas sensors producing signals when confronted with volatile compounds and an appropriate pattern-classification system [80]. At present, most of the EN are based on semiconducting metal oxides or conductive polymers gas sensors. However, other types of sensors including electrochemical, piezoelectric and optical sensors are also used [81]. Although not precisely being gas sensors, mass spectrometry and gas chromatography based EN provide an interesting alternative to the classical EN and will be presented briefly later.

The sampling system includes a chamber that hosts a sensor array mounted on a PCB card and a sample flow control unit (miniature membrane pump, flow meters and valves). Analytical signals received from sensors arrays are processed in three steps: data pre-treatment, feature extraction and dimension reduction, and pattern recognition algorithms.

EN found applications in many fields: food freshness and quality control (e.g., wine authenticity) [82,83,84,85], agriculture [86,87], disease detection (e.g., cancer, tuberculosis) [88], drugs detection (e.g., cannabis) [89], and security (e.g., fire warning, detection of explosives) [90,91,92]. EN are also increasingly used to monitor the indoor and outdoor air quality [34,39,93,94,95].

### 4.2. Electronic Noses Based on Gas Sensors

#### 4.2.1. Transducers for Multi-Arrays

**Figure 3:**
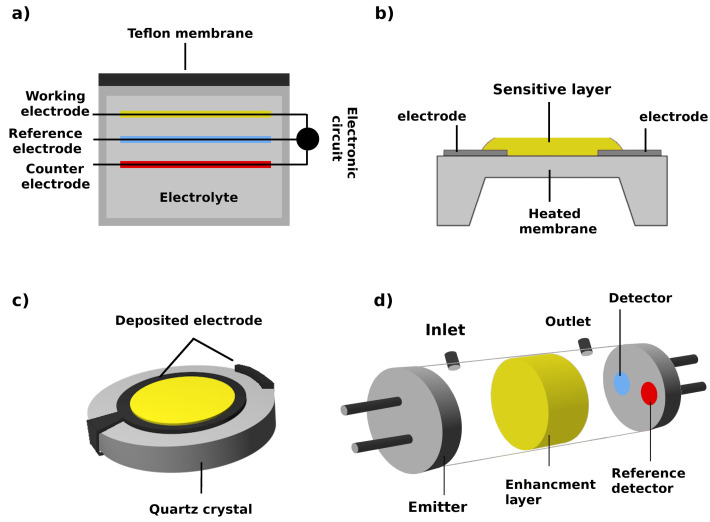
Principles of detection used for chosen gas sensors applied in e–noses: (**a**) electrochemical, (**b**) chemiresistive, (**c**) piezoelectric, (**d**) optical.

Gas sensors are transducers that transform chemical interactions between a sensitive layer and volatile molecules into an electrical signal. Many methodologies can be used for chemical sensing of gaseous compounds including chemiresistive, electrochemical, optical and piezoelectric sensors (Figure 3).

Electrochemical principle of detection is presented in Section 3.3 (and on Figure 3a). Chemiresistive gas sensors (Figure 3b) have a relatively simple configuration. The sensitive layer is deposited between two electrodes or on top of an interdigitated electrode. The interaction between the sensitive layer and the target gas molecules leads to resistance change of the sensor, mostly through the exchange of charge carriers [95].

Piezoelectric (QCM-Quartz Crystal Microbalance or SAW-Surface Acoustic Wave) sensors makes use of the piezoelectric property of the quartz crystal, which oscillates under an applied voltage across two gold electrodes, one of which is covered with the sensitive layer (Figure 3c). Its resonant frequency changes upon mass loading induced by the gas adsorption on the modified electrode surface [96]. SAW operate at 50-1000 MHz while QCM at 5-30 MHz. SAW devices are more sensitive but also more unstable and require a high-tech control set-up.

An optical sensor consists of a light source, a sensing platform, light waveguides and a light detector (Figure 3d). The sensing mechanism rely on the interactions of light and the sensing layer before and after exposing them to the target gas molecules. Alteration of the light intensity or shift in the wavelength of the light are the basic changes that can occur in the presence of the analytes of interest [97].

#### 4.2.2. Recent Advancement in Gas Sensing Materials

The sensitive layer can be made from various materials including semiconducting metal oxides, carbon nanomaterials, conductive polymers, molecularly imprinted polymers, biomolecules. Thanks to theirs electrical properties the first three of the listed above materials are usually applied for the development of gas sensors based on chemiresistive principle of detection.

Semiconducting metal oxides (e.g., SnO_3_, ZnO, WO_3_, CuO) have been more widely used to prepare gas sensors than any other class of materials [95]. Metal oxides can be fabricated in form of mico- or nanograins and deposited on a sensing platform as thick- or thin-films. The effect of grain size and film thickness on gas sensing has been investigated by many researchers and it may be concluded that the optimal performances are usually achieved with a thick film of lightly sintered nanocrystalline, porous materials [98]. Gas sensing properties of the metal oxide sensitive layers can be further tuned by using a combination of different metal oxides, addition of metal catalysts or application of hierarchical micro- and nanostructures [99,100,101].

Conducting polymers (e.g., polypyrrole, polyaniline, polythiophene, etc.) are easy to synthesize by various chemical and electrochemical approaches and their molecular chain structure can be modified conveniently by copolymerization or structural derivations leading to materials with new gas sensing properties [102,103]. Similarly to metal oxides, conducing polymers can also be prepared in form of nanoparticles, nanofibers, nanotubes, etc. However, so far, no spectacular improvements of sensing responses have been noticed with these nanostructures as compared to the thin film based sensors [104]. Another approach to improve sensing performances of devices based on conducting polymers is to combine them with other materials (metal, metal oxides, carbon nanostructures or even other polymers) [105,106].

Carbon nanostructures (e.g., carbon nanotubes, graphene) have been intensively studied for gas sensing applications recently. Theirs unique electrical properties can be further enhanced by decorating carbon nanotubes or graphene sheets with nanoparticles made of noble metals (e.g., Pt, Pd, Ag) [107] or metal oxides (e.g., SnO_2_) [108]. However, nanocarbon-based commercial gas sensors are yet to come as there is still a place for the improvement of gas sensing properties and reduction of production costs of these nanostructures [109,110].

Molecularly imprinted polymers (MIPs) are materials with custom-made binding sites complementary to the target molecules in shape, size and functional groups. Therefore, these materials are tailored at the synthetic level so that the selectivity of the designed sensor is oriented toward desired chemicals. However, it is hard to control the thickness of the MIPs films and thus, gas sensors based on these materials use usually QCM as transducer. The QCM appears a straightforward approach since it measures directly the “mass” adsorbed onto the microbalance [111].

Biomolecules, e.g., peptides and DNA also offers a promising option for the development of gas sensitive materials. The use of peptides is in a more advanced status since they represent the “natural” extension of the olfactory receptors. However, more studies are necessary to assess the long-term stability of devices based on these materials in real conditions [112]. Biomolecules are mostly used for the development of optical or QCM type gas sensors.

#### 4.2.3. Advantages and Disadvantages of Different Type of Gas Sensors

Due to theirs mature production technology, suitability to wide range of gases and low price, the main type of sensors applied in e-noses are chemiresistive metal oxide sensors (MOS). Today, several companies, such as Figaro, Sensirion or SGX Sensortech offer this type of devices. The MOS sensors operate at high temperatures (up to 500 ℃) and thus, they consume relatively high amounts of power: 100 mW per an ordinary sensor. However, with the advancement in electronic devices miniaturization, the size of MOS sensors become smaller through the years: from pin-type to MEMS patch-type ones and thus, energy requirements decreased to tens of mW. Application of multi-walled carbon nanotubes allowed further decrease in power consumption to only 1.05 mW [113]. The MOS sensors are sensitive to humidity changes and can be easily poisoned by high concentrations of volatile fatty acids or sulfur compounds [114]. The detection limit of most of the commercially available MOS sensors are at ppm levels and thus, they cannot be used for applications where analyzed molecules are at lower levels. Two ways to improve the sensitivity of these devices is investigated: development of sensors with lower detection limits by designing new sensitive layers as mentioned above and/or equipping the e-nose with an enrichment technology.

The production process of chemiresistive conductive polymers sensors (CPs) is not as mature as MOS ones, but this kind of devices can detect a wide range of gases without heating of the sensitive layer. This endows these sensors with low energy consumption and simple device configuration. They are also resistant to sensor poisoning but, in the same time, are sensitive to humidity and temperature changes. Other disadvantages, namely lack of selectivity, reversibility and stability of these sensing layers has been noted as well. One of the potential interesting approaches to improve CPs sensors is to prepare a hybrid material made of both: conducting polymers and metal oxides. The hybrid material is able to overcome the limitations of their single counterparts such as poor selectivity of conducting polymer and high working temperature of metal oxide, and hence, promotes an effective gas detection [105,106]. Although few commercial e-noses contain arrays based on conducting polymers (e.g., Cyranose from Sensigent), individual CPs are not available on the market.

Chemiresistive carbon nanomaterials sensors (CNs) usually operate at room temperature and thus, consume little amounts of power. Other advantages of CNs sensors include small size, long working life, ultra-high sensitivity, fast response and recovery times. Current studies focus on further improvement of CNs sensitivity, as well as their selectivity and stability. With the development of microelectronics technology, the cost of device manufacturing will drop and thus, the commercialization of sensors based on carbon nanomaterials will be more profitable [115].

The electrochemial sensors operate at room temperature, have low power consumption, long lifetime and are very robust. Moreover, they are not humidity sensitive and are suitable for toxic gases detection (e.g., H_2_S, NH_3_, CO, NO_x_). Electrochemical sensors are usually highly selective and thus, less compatible with the operation principles of the EN [116]. Nevertheless, electrochemical sensors are commonly used for construction of electronic tongues dedicated for the analysis of liquid samples [117,118].

Piezoelectric (SAWs, QCMs) are able to detect various gases at ppb levels in less than 10 s [119]. Besides, diverse sensing materials can be deposited on gold electrodes which increases the range of measured gases and thus, applications of these sensors. The SAWs and QCMs can be prepared with the MEMS technology which decreases their size. This allows to shorten the response and recovery times but, from the other hand, increases their instability, caused by the increased surface-to-volume ratio [120]. There are only a few commercially available devices based on this detection technique (e.g., Biolin Scientific, Quartz Pro) and usually sensitive materials are deposited on electrode on demand which makes these sensors relatively expensive.

Optical sensors can measure the concentration of a specific gas in gas mixtures with high sensitivity and selectivity. However, they are relatively expensive, difficult to miniaturize and exhibit low portability due to delicate optical and electrical components [121].

The advantages and disadvantages of gas sensors used in EN are summarized in Table 5.

### 4.3. Electronic Noses Based on Mass Spectrometry and Gas Chromatography

Mass spectrometers can be used together with chemometric programs to obtain a fingerprint of a particular odor and to proceed to classifications. After injection of volatile mixture an MS pattern is created. Each mass to charge ratio (m/z) acts as a sensor that detects any molecule or fragment with that particular ratio. In this way, an MS-based EN has potentially hundreds of sensors [123]. A big advantage of this system over gas sensors is that it uses a very well-known technology. The stability, reproducibility and sensitivity of mass spectrometers are well established. This offers a solution to some problems associated with classical gas sensors such as sensor poisoning or baseline drift. The MS-based sensors are also not sensitive to environmental changes (i.e., temperature and relative humidity) [85]. However, due to their large size MS electronic noses are less suitable for on site applications than EN based on classical gas sensors. Besides, they are expensive, consume high amounts of power and their construction is complicated [124].

The MS-based EN can be coupled with traditional gas chromatography. However, it is possible to skip this step and inject the sample directly to the mass spectrometer’s ionization chamber [125]. Interestingly, multi arrays made of classical gas sensors can be combined with gas chromatography as well (e.g., zNose from Electronic Sensor Technology) which increases applications of this type of devices.

EN based solely on flash/fast chromatography present yet another approach for the sensing of odorous mixtures. The Hercales e-nose from Alpha M.O.S is made of two columns with different polarities (and two FID detectors). Therefore, two different chromatographs are obtained during a single analyze. Although most of the available papers present applications of this device for food quality investigations [126,127,128], some studies suggest that it can be used for the monitoring of air quality in vicinity of municipal landfills or evaluation of air quality in the neighborhood of the petroleum plants [129,130,131]. Similarly to MS e-noses, the GC-based solutions are expensive, consume high amounts of power and their construction is complicated. Additionally, they require gas carrier gas and the analysis time is relatively long. For these reasons, GC based EN are not foreseen for on site applications. The advantages and disadvantages of GC and MS-based EN are summarized in Table 5 together with classical gas sensors.

### 4.4. Data Processing

#### 4.4.1. Data Pre-Processing

**Figure 4:**
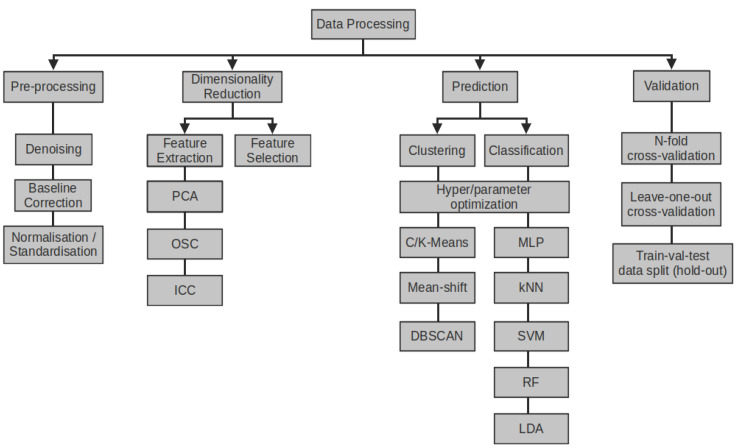
Summary of data processing methods.

Data processing methods are summarized on Figure 4. The dimensionality reduction, signal feature extraction, and data re-scaling methods are generally applied during EN data exploration and pre-processing.

The data collected by the EN may consist of several, several dozen or even several hundred variables that may be correlated to a greater or lesser extent [132]. Mutual correlation arises in the case of sensitive layers giving similar responses to the tested VOCs or results from the influence of environmental factors. This effect is usually considered as undesirable because too many dimensions make the data mining process difficult, can significantly extend the time of calculations and cause difficulties in achieving the target success rate when classifying signals. For this reason, dimensionality reduction methods such as Independent Component Correction (ICC) [133], Orthogonal Signal Correction (OSC) [134] or Principal Component Analysis (PCA) are used as first data preparation step. In the PCA method, which seems to be the most widely used method nowadays according to [85], the original data space is transformed in order to produce a new set of uncorrelated variables ordered by reducing variability. This allows to select only those sensors or those signal features that will effectively and significantly affect the classification results. PCA or OSC can be also used for detection of outliers [135], detection of sub-classes in groups of objects [136], classification or as EN drift correction method [137,138].

The aim of a feature extraction methods [139] is to simplify data analysis by extracting robust information from the sensor response. Extracting signal features may also be necessary in some rare cases. Zhang et al. [140] gives an example of an e-nose for the detection of flammable liquids where the measurements were limited to 10 s and the response stabilization was not possible. Feature extraction methods can be divided into three groups: methods that allow to describe a signal using simple scalar values, methods based on curve fitting, and methods based on signal analysis in the frequency or time domain. The first group of methods focuses on extraction of piecemeal signal features from sensor response such as: min/max, difference, fractional difference, primary derivative, secondary derivative, adsorption slope coefficient or area under the response curve [141]. Methods based on curve fitting fit a specific e.g., expotential or polynomial models to the response curves and extract a set of fitting parameters as the signal features [141,142,143]. The frequency or time domain analysis methods are based on signal transforms such as Fast Fourier Transform [144,145] or the Discrete Wavelet Transform [146,147,148] and are mainly applied to signals recorded over a long period of time or signals containing cyclical variation. Using features instead of raw signal measurements can help eliminate some additive errors [141] and compensate for the temperature influence on the sensors [149]. Additionally, some of the above mentioned features can be interpreted as physical parameters related to the reaction kinetics. For example, the signal slope coefficient (primary derivative) may represent the rate of the reaction of sensors responding to analytes and secondary derivative may represent the acceleration of the reaction, etc. [140,141].

The normalization and the standardization are the two primary scaling methods used in machine learning. Normalization typically re-scales the values into a range of [0,1]. Standardization typically re-scales data to have a mean of 0 and a standard deviation of 1 (unit variance). Both transformations allow to keep signal responses from various sensors of an e-nose at the same magnitude level. This step is particularly advised for pattern recognition or classification methods based on Artificial Neural Networks (ANN) as they tend to learn from signals differences [150]. In a case of large differences in raw signal magnitudes, ANN will tend to favor the sensor or sensors with the highest numerical values. It is proven that normalization improves precise identification of the odor concentration and helps to reduce the calculation error of stoichiometric recognition [141].

#### 4.4.2. Classification Methods

The modern approach to the signal classification is dominated by data mining methods, which are divided into supervised and unsupervised methods. The main advantage of unsupervised methods is lack of a learning stage and no need to describe the dataset, which in the case of large number of data would be an expensive, laborious, and time consuming operation [151,152]. PCA and cluster analysis methods such as DBSCAN [153], Mean-shift [154] or k-means clustering [155] are categorized as unsupervised methods. A cluster is a subset of the initial dataset whose data points have one or more features in common which results from sensors reacting to the same volatile substance under similar conditions of temperature and humidity. From computational point of view these features may be: location in the same dense areas of the data space, small distances between cluster members or membership of the same statistical distributions. Two issues limit the applicability of unsupervised methods as automatic classification algorithms. Firstly, clusters are not usually known beforehand, and are calculated by the algorithm starting from random initial locations. This forces the expected number of clusters to be specified as an integer value, before computation begins. In some cases, the optimal number of clusters can be computed or is defined a priori. The second problem is the lack of an unambiguous method or metric allowing to determine the accuracy of the clustering without using the descriptive information provided by an human operator. For this reason, unsupervised methods are mostly used as pre-processing methods [85].

**Figure 5:**
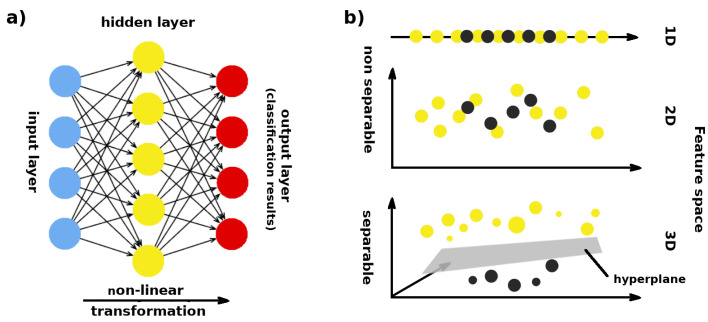
Simplified visualisation of: (**a**) multi layer perceptron, (**b**) support vector machine kerneling.

In order to automate e-nose data classification supervised methods such as: Linear Discriminant Analysis (LDA), Linear Least Squares regression (LLS) and nonlinear Support Vector Machines (SVM) classifiers, Random Forest (RF), k-NN classifiers, and artificial neural networks (ANN) are used [85]. Supervised methods tend to produce better results and provide the opportunity to generate a metric such as confusion matrix [156] that can be used to compare or to optimize the results. Of all the various ANN architectures the most used is the Multi-Layer Perceptron (MLP) trained using a cost minimisation function known as the backpropagation algorithm [157]. The MLP (Figure 5a) consist of one input layer for e-noses signals or signals features, one or more “hidden” layers of coefficients (weights and biais) which are interconnected to the hidden layer inputs and outputs and one output layer which holds the classification results. During a finite number of iteration the MLP coefficients are modified (trained) until the error between input labels and target classes is minimized.

The SVM working principle is different. The SVM creates a line or a plane which splits data points into classes with the greatest possible margin between the plane and any point within the training set, giving a greater chance of new data being classified correctly. Data points are progressively mapped into higher and higher dimensions (Figure 5b) in a process known as kernelling [158], until a hyperplane can be formed to segregate them. Both ANN and SVM methods are widely used in research [38,143,159,160]. According to some authors, the SVM seems to outperform ANN [161], however, it should be remembered that the results of such comparative studies strongly depend on the data, the configuration, parameter tuning and the choice of ANN architecture. For this reason, when choosing an appropriate method, we should rely on the results of experiments rather than on bibliographic premises.

#### 4.4.3. Challenges in Data Processing

From computational point of view the biggest challenge is to achieve the best accuracy and the lowest level of false positive and false negative detections. For EN operating in a controlled environment e.g., in a lab bench, a 100% success rate in samples classification is often achieved [162,163]. Indeed, in laboratory conditions it is possible to calibrate e-nose with a wide range of gas concentrations and thus, increase the performance of the system [164]. The interferences from non-target odors present in real samples is also limited. In real live experiments such high accuracy is never reached [165,166,167]. An obvious solution to this problem is to increase the number of samples covering wide areas, under different meteorological conditions [166] or increasing the number of sensors [132], which makes the data analysis more difficult. Although some authors were able to constitute a large (18,000 samples) data set of gas sensor arrays responses in laboratory conditions [168], the size of a typical data set intended for e-nose training reported in publications is rather limited to 100–500 samples approximately [139,167,169,170,171]. The number of samples collected in outdoor experiments is sometimes critically low (~50) [172]. Therefore, the success rate of 100% in outdoor conditions published by some authors [173] may be due to a very low number of samples collected.

Another challenge is related to machine learning, which in most publications is presented in a simplified manner. A critical step in ANN classificator setup is the determination of right activation function and the optimal number and size of hidden layers. The only guideline in this matter is the knowledge that three-layered networks have sufficient computational degrees of freedom to solve any classification problem [143]. In the case of SVM classification, the most critical step is choosing a suitable kernel of SVMs for a particular application. Various applications need different kernels to get reliable classification results [174,175]. At the moment, there is no framework that automates the selection of the best architecture or kerneling function. So the success of this crucial stage depends heavily on the authors perseverance and programming skills. Only in a few publications we can find examples of a detailed description of machine learning process with: train and validation loss curves [169], information about sampling strategy adopted [166] or training, validation, and test sets for unbiased evaluation of models [139]. In other cases, it is usually impossible to extrapolate published results to new problems.

### 4.5. Commercially Available E-Noses

A summary of some of the most widely used electronic noses with manufacturers, models, technological basis and data processing methods are listed in Table 6.

Commercially available EN based on semiconducting metal oxide can be purchased (among many others) from Airsense Analytics GmbH (www.airsense.com, accessed on 8 February 2022). The company proposes the PEN series of e-noses (iPEN, PEN2, PEN3) which contain an array made of 10 metal-oxide semiconductor (MOS) gas sensors. The measuring cell can be linked with an adsorbent trapping unit or a headspace auto sampler for laboratory analyses. The company offers also other devices: Olfosense and GDA (GDA-FR, GDA-X). The first one combines MOS technology with PID detectors and electrochemical sensors. The GDA units contain 2 MOS sensor, a PID detector, an electrochemical cell and ion mobility spectrometer (IMS). The PEN and GDA series are portable devices that found applications in environment investigations and security, respectively. The Olfosense aims to set up a Network for Air Quality Monitoring where multiple devices are interconnected, and users keep their eyes in real time on environmental parameters, i.e., odor concentration (ou/m^3^),VOCs, H_2_S and NH_3_ concentrations (from ppb to ppm).The device is provided with a dispersion modelling. Similar approach is proposed by Odotech (www.odotech.com, accessed on 8 February 2022), SACMI (www.sacmi.com, accessed on 8 February 2022) or RubiX (www.rubixsi.com, accessed on 8 February 2022) company.

Through the years, Alpha M.O.S (Toulouse, France) developed few EN based on metal oxide semiconducting sensors. Some of them like FOX 2000, 3000, 4000 were based solely on MOS sensors and others (RG BOX, Prometeus) were equipped additionally with electrochemical and PID sensors or mass spectrometry detector. These devices are no longer on the market. Indeed, few years ago the company changed completely direction and released an EN (Heracles Neo) based on ultra fast gas chromatography (www.aplha-mos.com, accessed on 8 February 2022). This e-nose has been described above.

The conducting polymer technology is used in Cyranose 320 provided by Sensigent (www.sensigent.com, accessed on 8 February 2022). The device contains a total of 32 individual conducting polymer–carbon black sensors. The piezoelectric effect is used for example in the SAGAS e-nose from Forschungszentrum Karlsruhe (www.kit-technology.de, accessed on 8 February 2022) or ZNose series (ZNose 4200, 4300, 7100) from Electronic Sensor Technology (www.estcal.com, accessed on 8 February 2022). The last one is coupled with ultra-fast chromatography.

## 5. Application of the Electronic Noses for Monitoring of Mechanical–Biological Treatment of Waste: Analytical Measurements

### 5.1. Composting

Composting is an aerobic process that is used to convert organic waste into agriculturally useful products. Composting can be divided in two phases: high-rate composting phase and a curing phase [176]. An intense microbial activity occurs during the first phase leading to the decomposition of the most biodegradable material. The rest of the material is slowly transformed into humic substances during the second phase. The first phase consumes large amounts of oxygen which may lead to anaerobic conditions and thus, a correct air management (air supplier) is necessary to achieve optimal composting conditions.

EN are promising tools that allow a constant monitoring of the composting process, assessment of the compost quality, maturity and its biological stability. Romain et al. [177] used an emission chamber equipped with a home-made EN device composed of 7 metal oxide sensors for monitoring of the composting process of household wastes during 60 days. The humidity, temperature and air flow in the chamber were recorded as well. The EN was not set to determine individual chemicals, but whole chemical families (sulphur compounds, alcohols, nitrogen compounds, aldehydes, ketones, esters, acids, furans, dioxins, ethers, terpenes, chlorinated compounds and hydrocarbons). The alcohol family prevails during the whole composting time except in the earlier stages when aromatic and aliphatic hydrocarbons are the most abundant. A peak of nitrogen compounds and carboxylic acids appears at day 17th and a high increase of the ketones and furans occurs the last days of composting. The peak at day 17th is due to the absence of proper aeration: there was no turning of the pile since 5 days. Ketones and furans are released due to chemical processes which indicates the end of the high-rate composting phase and beginning of the curing phase. Therefore, the presence of these species at the end of the experiment is not surprising.

Lopez et al. [71] used commercially available PEN3 e-nose based on metal oxide sensors for the in-situ assessment of compost stability and maturity. A total of 7 different composting piles from a commercial facility located in Spain were investigated. Development of anaerobic conditions was confirmed in some of the experiments as oxygen levels dropped to c.a. 10% leading to changes in volatile organic compounds emissions as it was noticed with the EN measurements. In support of this argument combustible gases were also detected in all these samples with NDIR sensor. Therefore, the EN device is suitable for the classification between aerobic-anaerobic conditions of the composting pile and thus, indication of its stability and quality. Moreover, changes in the chemical composition of gaseous samples with the composting time were recorded and appropriately trained e-nose was used later on to estimate the compost maturity.

The PEN3 e-nose was also used for the assessment of the biofiltration efficiency of the composting gases [178]. The pile was fed with kitchen wastes or shredded pruning waste. The air from the pile was extracted by an exhaust fan and distributed upstream to a set of 12 pilot-scale open-top biofilters. The compost gases from the biofilters inlet and outlet ports were monitored for 20 days by the EN and the total VOCs were analyzed by a photoionization detector (PID). The biofiltering process of the emitted gases reached total VOCs removal efficiency greater than 90% as identified by PID. The EN could identify qualitative differences among the biofilter output gases related to its nature and particle size. Sensors detecting sulphur containing-compounds were especially discriminating. The EN could also be used to quantify total VOCs content in air samples during the composting and biofiltering trial.

Gutierrez et al. [35] compared 3 complementary approaches to monitor odorants and odors (dynamic olfactometry, GC-MS and EN). The odor source was a green waste compost at different maturity stages located in the south of Belgium. The EN made of 6 Figaro MOS sensors was capable of identifying some chemical families emissions and some activities such as turning steps, whereas the GC-MS detected each individual chemical. Odor concentrations (ou/m^3^) in relationship with these emissions were determined by dynamic olfactometry. The olfactometry indicated an increase of the odor concentration during the first days of the composting process. The GC-MS measurements shown that odorants responsible for the perceived odor belonged to the following chemical families: terpenes, organic acids, ketones, aldehydes and alcohols like D-limonene, butanoic acid, thujone, hexanal and 2-butanol, respectively. E-nose data were linked to the chemical composition and to the odor concentration.

Although most of the EN systems dedicated for the compositing processes monitoring are based on the MOS sensors, other types of sensing devices have been used as well. Lieberzeit et al. [179] designed e-nose based on 6 QCM sensors coated with molecularly imprinted polymers (MIP). They enable quantitative monitoring of the most relevant volatile organic compounds, i.e., esters, alcohols and terpenes emitted form the compost process of grass and pine. During the composting authors observed concentrations of up to 250 ppm of esters, 700 ppm of alcohols, 250 ppm of terpenes directly on-line and validated the data off-line by GC-MS. The EN gave also direct insight into the differences between the two composting batch types. During grass composting larger amounts of alcohols are emitted whereas relative content of terpenes is twice as high for pine composting.

### 5.2. Anaerobic Digestion–Biogas Formation

Anaerobic digestion is a biological process in which the organic matter is converted by specific microorganisms into biogas (mainly CH_4_ and CO_2_) that can be used for electricity generation. At the beginning of the process the matter is decomposed to mono- and oligomers (amino acids, long-chain fatty acids and saccharides), then the fermentation of the volatile fatty acids appears (mainly acetic acid), followed by gases (H_2_, CO_2_) and finally CH_4_ production. The parameters affecting the status of anaerobic digestion are mainly associated with changes in plant feeding and environmental fluctuations, i.e., temperature [180]. The demand for online monitoring and control of biogas process is increasing, since it can improve process plants stability and economy.

The concentration of some compounds in the gas phase changes when the organic overload (ORL) appears in the reactor. The ORL leads to process disturbance and thus, influences the effectiveness of methane production. Adam et al. [181] developed an EN composed of six commercial MOS gas sensors for early detection of organic overload in the reactor. The e-nose was set to detect CH_4_, H_2_S, H_2_, alcohols, alkanes, alkenes and ketones. Hotelling’s T^2^ value and an upper control limit using stable digesters as a reference set were used as indirect state variable for early detection of process disturbance caused by organic overload. The EN could detect organic load variations and it was also able to monitor process disturbances and recovery periods. Moreover, in some cases the e-nose technology appeared to be more efficient than the monitoring of methane content in the biogas or pH measurements of the liquid phase. Furthermore, Adam et al. [182] investigated the use of the above mentioned EN for online anaerobic reactor state monitoring at the pilot-scale and then at the full scale level. At the pilot-scale level, the e-nose observations present a good agreement with the reactor state. At the full-scale level, the device provided warnings of the major disturbances in the reactor but two slight disturbances were not detected and it gave one major false alarm. This work showed that gas phase relation with anaerobic process should be deeper investigated, as an EN could indicate the reactor state, focusing on the gas phase. Similar conclusions can be drown from Costa et al. [183]

Orzi et al. [184] investigate the correlation between microbial activity (i.e., biological stability measured by aerobic OD_20_ test and ABP anaerobic tests) and odor emissions from municipal solid waste during anaerobic digestion in a full-scale treatment plant considering the three stages of the process (input, digested and post-digested waste). Authors used complementary approaches for the measurement of odor impact (olfactometry) and characterization of the different groups of VOCs responsible for that odor (GC-MS and PEN2 e-nose from Airsense). As expected the odor reduction due to the acquirement of biological stability was accompanied by a change in the organic molecules composing the gaseous phase in the studied wastes. Moreover, principal component and partial least squares analyses applied to the EN and GC-MS data sets gave good regression for the OD_20_ vs. the EN and OD_20_ vs. the GC-MS data. Therefore, OD_20_ reduction could be used as an odour depletion indicator. Presented above applications are summarized in Table 7.

## 6. Application of the Electronic Noses for Odor Impact Assessment– Sensory Measurements

Electronic noses can be trained to determine odor concentrations (ou/m^3^) or discriminate odor sources. Romain et al. [185] used a home-made EN made of 7 Figaro MOS sensors for the monitoring of odor concentration inside a compost hall. Measurements were validated with dynamic olfactometry. Authors suggested that measurements with EN at odor source can be applied together with dispersion models in order to asses the odor impact in the vicinity of the facility. The same authors [186] studied also the influence of sensor drift in time on the odor source discrimination capability of an e-nose composed of 12 Figaro MOS sensors. Authors reported that classification accuracy decreased from 98% at the beginning of the tests to only 20% after three years. Authors suggested to solve this problem by additional training sessions during the monitoring period. Another approach proposed by Capelli et al. [187] requires an internal calibration system that allows to estimate the sensor drift daily and to compensate it with suitable algorithms.

Discrimination of odor sources was also performed by Sironi et al. [188] who applied two EN developed earlier in their laboratory [189]. First one was installed at the composting plant territory and second one inside a house situated 300 m from the facility. During a 4 days trial the e-nose placed inside the house detected the presence of odors coming from the composting plant for about 7.8% of the total monitoring duration. The results were compared with odor complaints from inhabitants, obtaining a correspondence of 72%. Moreover, 86% of detected odorous events corresponded to the open air storage of the waste screening overflows heaps, which was therefore identified as the major odor source of the investigated composting plant. Later on, the same authors investigated again the site but the monitoring time was increased from few days to few weeks [190]. The purpose of this work was to evaluate which features had to be extracted from the sensor responses curves in order to optimize the correlation between odor classification performed by an EN and odor reports from inhabitants. During this study, the variability of humidity content in analyzed air turned out to be a critical factor in the use of EN for environmental air monitoring. The problem was solved later one [189]. Indeed, electronic nose was equipped with a specific humidity regulator that allows the instrument to be used in the field under variable meteorological conditions. Improved e-noses were installed at several locations (waste treatment plants, oil mill and in the surrounding urban area) and demonstrated odor sources classification accuracy of 88%. In the same time, older version of the e-nose correctly classified 82% of measurements during a 10 days trial. Authors described also an experimental procedure for the definition of minimum performance requirements for environmental odor monitoring, which is a first step towards standardization of the specific case of electronic nose application for environmental odor monitoring [191].

Indeed, the use of EN in the field is very challenging, especially in variable weather conditions. Nicolas et al. [33] placed a network of 5 e-noses comprising each 6 metal oxide sensors (Figaro) in the close surroundings of a compost facility in Belgium. The EN was trained to estimate odor concentration (ou/m^3^) and classify odor sources into five possible categories odor-free atmosphere, engine exhaust gases, green compost, bio-drying or fresh waste emissions. Moreover, according to the wind direction, the responses of e-noses placed in the right wind sector were used to assess the maximum downwind distance of odor perception. A simple validation test was carried on by comparing the detection of odor events by the network and the subjective feeling of field observers placed near the electronic noses. About each event detected by the network was validated by field observer. Then residents in the neighboring villages were appealed to regularly provide their estimation of the odor annoyance, according to a procedure described by Nicolas et al. [192]. In 17 cases out of 21, the network detected an odor event when residents identified an odor coming from the waste treatment plant. For 10 cases among these 17, the resident concerned was included in the perception zone predicted by the e-nose network. The 7 remaining cases corresponded to unstable atmospheric conditions. Authors concluded that a network comprised of a higher number of e-noses could compensate for the variable weather conditions.

An interesting work was described by Giungato et al. [165] who tested two commercially available sensors (PEN3 and Cyranose320) for odor source discrimination capabilities. Three types of odors were investigated: biogas, a by-product of mechanical treatment of municipal solid wastes, and a sludge pressed and dehydrated from treatment of urban wastewater. The MOS based e-nose was able to discriminate successfully 86.7% of samples, whereas the CPs only 53.3%. Surprisingly, the best results were achieved by choosing 4 MOS and 2 CPs sensors (93.3%). This work highlights how important is the selection of the adequate type and number of sensors used for a given application. Taking into account that described above investigations were performed using mainly MOS sensors (usually from Figaro), it is safe to say that at least in the field of odorous mixtures investigation in the vicinity of waste management plants there is still a lot of possibilities in terms of appropriate sensors selections.

The discriminatory possibilities of two types of EN in the distinction and classification of odorous samples collected around a municipal landfill was studied by Gębicki et al. [36,130]. One of the tested e-noses was the Heracles Neo from Alpha M.O.S based on ultra-fast gas chromatography technology. The second device was a home-made e-nose composed of MOS, PID and electrochemical sensors. Additionally, field olfactometry investigations were performed at places where samples were collected for analyses using both types of electronic noses. A correctness of classification of 94.8% and 84.4% was achieved for Heracles Neo and home made e-nose, respectively. These results corresponded to samples collected during spring period and thus, characterized by high odor nuisance. For lower odor concentrations observed in winter, the correctness of classification dropped to 87.5% and 71.9%, respectively. Authors concluded that comparing the price of both e-noses the home-made one exhibits a satisfactory level of correct results as compared to much more expensive Heracles Neo. As suggested by the same authors [193] the performance of e-nose based on gas sensors can be improved by implementation of more and more sensitive sensing arrays. Presented above applications are summarized in Table 8.

## 7. Conclusions and Perspectives

Electronic noses are promising tools for the control of waste treatment process and odor impact assessment in the waste management plants. Multiple examples presented in this review have shown that once adequately trained, EN can be used successfully for quantitative and qualitative measurement of odorants in the analyzed samples as well as for odor concentration measurements and odor sources discrimination. Comparing to other methods, analysis with electronic noses allows the measurements to be run continuously. However, EN should not be treated as competitive method for the well established sensory and analytical techniques. Indeed, current trends clearly stands that maximum information on the odorous samples investigations is achieved when olfactometry, GC-MS and e-noses are used as complementary approaches to a given problem.

Bibliography presented in this review discusses also some critical aspects connected with the development of the EN devices. Concerning the sensors, several studies have highlighted the problem of stability towards temperature and humidity variations, as well as sensor response drift over time. Possible solutions to these problems using appropriate data processing methods were presented in this paper showing that the EN devices require time-consuming calibrations and re-calibrations and/or sophisticated and complex technology in order to produce accurate and reliable results. Despite these problems a few commercial e-noses dedicated for the environmental monitoring exists. However, mainly due to the problems associated with sensors stability the warranty provided by their manufacturers is usually very short (i.e., 12 months) and given the price of these devices–not satisfactory. The malfunctioning sensor in most cases can not be replaced by a new one due to the poor reproducibility of the gas sensors’ manufacturing.

Actually, there are several trends in order to improve sensing performances of existing EN, including development of new gas sensors. Although nanotechnology can provide a solution to the sensitivity issues, the long term stability of gas sensors based on nanoparticles is still an issue.

Future trends regarding the use of EN in the environmental analyses shall not only focus on the development of new sensors or data processing methods, but also concentrate on better adjustment of these instruments for outdoor conditions, especially for long–term monitoring applications and adaptations on mobile platforms and on portable devices. Indeed, the possibility of drone assisted measurements using EN are currently intensively examined [194].

Another important issue limiting the use of EN for environmental applications is the lack of specific regulation for their standardization. EN are relatively complex instruments, and their development give rise to a number of degrees of freedom, regarding mainly the choice of gas sensors, the training and data processing methodology. Therefore, the standardization of the EN devices and procedures for their correct utilization is a necessary step for their diffusion.

## Figures and Tables

**Table 1:** Chemical composition of odorous mixtures from different stages of waste management.

Stage of the Waste Management	List of Detected Substances	References
Collection and transport	ethanol, dimethyl sulfide, methyl mercaptan, dimethyl disulfide, propylene, ethyl acetate, NH_3_, methacrolein, benzene, toluene, ethylbenzene, methyl chloride and m-,p-xylene	[7,22,23,24]
Waste transfer stations	ethanol, methyl mercaptan, dimethyl disulfide, H_2_S, propanal, m,p-xylene, methacrolein, acrolein, NH_3_, benzene, toluene, acetaldehyde, acetic acid, and butyric acid.	[23,25,26,27]
MBT facilities	acetic acid, butyric acid, valeric acid, isovaleric acid, and dimethyl sulfide	[28]
Landfills	H_2_S, methanethiol, dimethyl disulfide, carbon disulfide, diethyl disulfide, benzene, NH_3_, ethyl acetate, ethylbenzene, p-ethyltoluene, n-hexane, 1,2-dichlorobenzene, trichloroethylene, styrene, m-xylene, toluene, p-xylene, acetone, methanol, n-butanone, acetic acid, and 2-octanone	[29,30,31]

**Table 2:** Odor measurement methods and their limitations regarding sensory and chemical detection.

Measurement Method	Chemical	Sensory	Continuous
Olfactometry	no	yes	no
GC-MS	yes	no	no
GC-O-MS	yes	partially	no
Single sensors	yes	no	yes
E-nose (training with GC-MS)	yes	no	yes
E-nose (training with olfactometry)	no	partially	yes

**Table 3:** Examples of recent applications of sensory measurement in the waste treatment plants.

Application	Methodology	Main Outcome of the Work	Ref.
Odor impact assessment	Dynamic olfactometry, “Operat Fb” dispersion model	Landfill was mainly responsible for odor nuisance caused by the waste management plants	[52]
Odor impact assessment	Dynamic olfactometry, CALPUFF dispersion model	Dispersion models are efficient tools for odor mitigation strategies investigation	[55]
Odor impact assessment	Dynamic olfactometry, CALPUFF dispersion model	Modeling choices may lead to a variance in the resulting odor concentrations	[56]
Odor impact assessment	Field olfactometry, CALPUFF dispersion model	Exposure to odor nuisance is an important factor in urban areas management and planning	[53]
Composting process monitoring	Dynamic olfactometry (supported by physical-chemical and respirometry measurements)	Dynamic olfactometry is a sufficient and simple method to assess compost stability	[57]
Monitoring of anaerobic digestion process	Field olfactometry (supported by GC-PID)	Field olfactometry can be used for both, odor impact assessment and monitoring of anaerobic digestion processes	[60]
(Bio)filtration efficiency assessment	Dynamic olfactometry	Organic filter presents higher deodorization efficiency than mineral one	[58]

**Table 4:** Examples of recent applications of analytical techniques for the waste treatment processes monitoring.

Application	Methodology	Main Outcome of the Work	Ref.
Monitoring of VOCs released during initial stages of waste treatment	GC-MS, calculation of OAV	The EBW proportion in waste is the dominate source of VOCs	[7]
Monitoring of VOCs released during composting of food, yard and paper wastes	GC-MS	Waste origin plays a crucial role on the chemical composition of VOCs	[72]
Monitoring of VOCs released during household composting of food wastes	GC-MS, physicochemical measurements, PCA	PCA applied to VOCs and physicochemical parameters is a sufficient tool for the monitoring of the composting process	[73]
Biofiltration efficiency assessment	Ammonia electrochemical sensor	Waste origin plays a crucial role on the biofiltration efficiency	[74]
Monitoring emissions of odorants released from waste biogas plants	Multi-gas detector (PID and H_2_S, NH_3_, CH_3_SH electrochemical sensors), calculation of OAV	Odorant concentrations and odor activity value can be useful tools for the control of technological processes	[75]
Monitoring of biogas generated from landfills	Internet of Things system equipped with gas sensors	Biogas content emitted from landfills may present dangers and sanitary risks	[76]

**Table 5:** Advantages and disadvantages of different sensor types used in e-noses based on [85,87,122].

Sensor Type	Advantages	Disadvantages
Classical gas sensors		
Chemiresistive metal oxide sensors	Suitable to wide range of gases Good sensitivity (ppm and sub–ppm) Long lifetime Short response time Mature technology production Low cost, Small size, Easy to use	Operates in high temperatures Vulnerable to poisoning Humidity sensitive Baseline drift
Chemiresistive conducting polymers sensing	Suitable to wide range of gases Operates at room temperatures Resistant to sensor poisoning Good sensitivity (ppm) Short response time Low cost, Small size, Easy to use	Temperature and humidity sensitive Limited sensor lifetime Poor selectivity, reversibility and stability Baseline drift
Chemiresistive carbon nanotubes and graphene sensors	Ultra-high sensitivity (ppb) Usually operates at room temperature Fast response and recovery time	Temperature and humidity sensitive Difficult to fabricate, expensive Poor reproducibility
Electrochemical	Power efficient and robust High selectivity Ambient temperature operation Suitable for toxic gas detection	Large size Not suitable to wide range of gases
Piezoelectric	Very high sensitivity (ppb) Diverse sensing materials Fast response and recovery times	Temperature and humidity sensitive Poor signal–to–noise ratio Complex fabrication process
Optical	High sensitivity, selectivity and stability Fast response and recovery times Insensitive to environment change	Difficulty in miniaturization High cost and high power consumption Low portability
MS and GC based e-noses		
MS	Insensitive to environment change High sensitivity, stability, reproducibility Resistant to sensor poisoning and baseline drift Well known technology	Expensive Consume high amounts of power Difficulty in miniaturization Complicated construction
GC	Insensitive to environment change Resistant to sensor poisoning and baseline drift High sensitivity, stability, reproducibility	Large and heavy Complicated construction Very expensive Require carrier gas Not foreseen for on site applications

**Table 6:** Examples of commercial e-noses.

E-Nose	Technology	Data Processing	Applications	References Accessed on 8 February 2022
AirSense Analytics-PEN	MOS	DFA, PCA, LDA, PLS and more	Environment, security and quality control (including: odor concentration)	www.airsense.com
AirSense Analytics-Olfosense	MOS, PID, EC, OPC	PCA, PLSR	Environment (including: odor concentration with dispersion modeling)	www.airsense.com
AirSense Analytics-GDA2	MOS, EC, IMS, PID	non defined	Hazardous gases, chemical warfare detection	www.airsense.com
Alpha M.O.S-Heracles Neo	Flash GC	PCA, DFA, PLS and more	Food control quality, new aroma development	www.alpha-mos.com
Applied Sensor-Air Quality Modules	MOS	PCA, PCR, LDA, ANN and more	Indoor air quality monitoring, diverse industries (food, chemical, textile)	www.applied-sensor.com
Aryballe-NeOse Pro	Optical biosensors	PCA	Diverse industries (automotive, food, beverage)	www.aryballe.com
Electronic Sensor Technology-zNose	SAW with flash GC	non defined	Healthcare, medical research investigations, security, outdoor air quality and environmental odor monitoring, diverse industries (food, beverage, chemicals)	www.estcal.com
KIT Karlsruher-SAGAS	SAW	ANN, PLS, LDA, Cluster, PCA	Indoor air quality, chemical industry	www.kit-technology.de
Odotech-OdoWatch	MOS	ANN, Cluster	Environment (continuous monitoring of odors and other gaseous contaminants with dispersion modeling)	www.odotech.com
RoboScientific Ltd.-Model 307	CPs	non defined	Plants and animals disease detection (including COVID-19)	www.robo-scientific.com
RubiX - WT1	MOS with optionally: EC, PID, OPC, NDIR...	PCA, LDA, PLS	Outdoor and indoor air quality, environmental odor monitoring (including: odor concentration withdispersion modeling)	www.rubixsi.com
Sensigent-Cyranose 320	CPs/carbon black	PCA, k-NN, k-means, SVM and more	Medical research investigations, outdoor air quality and environmental odor monitoring, diverse industries (food, beverage, chemicals)	www.sensigent.com
The eNose Company-Aeonose	MOS	non defined	Healthcare (cancer detection)	www.enose-company.com
SACMI-EOS Ambiente	MOS	PCA, DFA, LDA, ANN, PLS, SVM and more	Environment (including: odor concentration with dispersion modeling)	www.sacmi.com

CPs–conducting polymer sensors, EC–eclectrochemical sensor, GC–gas chromatography, IMS–ion mobility spectrometer, MOS–metal oxide gas sensors, MS–mass spectrometer, OPC–optical particle counter, PID–photoionization detector, SAW–surface acoustic wave.

**Table 7:** Examples of analytical e-noses applications in the waste management plants.

Application	E-NoseTechnology	AdditionalMeasurements	Ref.
Qualitative detection of VOCs during composting processMonitoring of the compost stability	Home made (7 MOS)	GC - MS	[177]
Compost maturity assessment Monitoring of the compost stability	PEN3 AirSense Analytics	NDIR, PID, GC-MS	[71]
Bio-filtration efficiency of the composting gases	PEN3 AirSense Analytics	PID	[178]
Correlation between odor concentration and chemical composition of VOCs emitted during composting process	Home made (6 MOS)	Olfactometry, GC-MS	[35]
Qualitative and quantitative detection of VOCs during composting process	Home-made (6 QCM)	Validation with GC-MS	[179]
Early detection of organic overload in the anaerobic reactor	Home-made (6 MOS)	NDIR, EC, sludge pH	[181,182]
Investigation of the correlation between microbial activity, odor concentration and VOCs emission during anaerobic digestion of wastes	PEN2 AirSense Analytics	Olfactometry, GC-MS	[184]

**Table 8:** Examples of sensorial e-noses applications in the waste management plants.

Application	E-NoseTechnology	Additional Measurements	Ref.
Monitoring of odor concentration inside a composting hall	Home-made (7 MOS)	GC-MS, dynamic olfactometry, field inspections, dispersion modeling	[185]
Discrimination of odor sources in the vicinity of a composting plant	Home made (6 MOS)	Olfactometry, citizens involvement	[188,190]
Monitoring of odor concentration and discrimination of odor sources in the close neighborhood of a composting facility	Network of 5 e-noses made of 6 MOS	Dynamic olfactometry, citizens involvement, dispersion modeling	[33]
Discrimination of odor sources	Network of 5 e-noses made of MOS	Dynamic olfactometry	[189]
Comparison of odor sources discrimination capability of two commercial e-noses	PEN3 and Cyranose 320 commercial e-noses	None	[165]
Comparison of odor sources discrimination capability of two e-noses	Heracles Neo and home-made e-nose based on MOS, PID and EC sensors	Field olfactometry	[36,130]
Odor concentration measurements in the vicinity of several odor sources (including landfills)	Home-made e-nose based on MOS	Field olfactometry	[193]

## Data Availability

The data that support the findings of this study are available from the corresponding authors upon request.
